# The potential role of miRNAs in therapy of breast and ovarian cancers associated with BRCA1 mutation

**DOI:** 10.1186/s13053-017-0076-7

**Published:** 2017-09-29

**Authors:** Agnieszka Strumidło, Sylwia Skiba, Rodney J. Scott, Jan Lubiński

**Affiliations:** 1 Pomeranian University of Medicine, Szczecin, Poland; 2 The University of Newcastle and Hunter Medical Research Institute, Newcastle, Australia; 3 Division of Genetics, Hunter Area Pathology Service, Newcastle, Australia; 4 Department of Genetics and Pathology, Pomeranian University of Medicine, Szczecin, Poland

**Keywords:** Breast cancer, Ovarian cancer, BRCA1, microRNA, Treatment

## Abstract

Germline variants within BRCA1 or BRCA2 genes account for approximately 25% of familial aggregations of breast-ovarian cancers. Low or no expression of BRCA1 in breast and ovarian cancers is associated with a good clinical response to treatment with platinum therapies and PARP1 inhibitors. Recent studies demonstrated that microRNAs - small non-coding RNAs, involved in the control of gene expression, can decrease BRCA1 expression by targeting the 3’UTR region of the gene. This article reviews reported relationships between various miRNAs, such as miRNA-9, miRNA-146a, miRNA-182 miRNA-218, miRNA-638 and the response to cytostatic drugs, mainly to platins and PARP1 inhbitors, for the treatment of breast and ovarian cancer associated with BRCA1 mutations.

## Background

Breast cancer is the most common malignancy in women. It is estimated that worldwide over 508,000 women died in 2011 due to breast cancer (WHO 2013) [1]. Ovarian cancer is the seventh most common malignancy. It was estimated that in 2012 about 152,000 women died of ovarian cancer in the world [2].

BRCA genes encode proteins responsible for double-stranded DNA break repair processes associated with replication. They are involved in the process of recognizing and orchestrating the removal of double strand breaks (DSBs) by homologous recombination (HR). If both copies of either BRCA1 or BRCA2 are mutated, HR fails to be initiated, resulting in genomic instability and consequently tumour initiation [3, 4].

Germline variants within BRCA1 or BRCA2 genes account for approximately 25% of familial aggregations of breast-ovarian cancer that are clinically manifested as hereditary breast and ovarian cancer syndrome (HBOC) [5, 6]. Low or no expression of BRCA1 in cancers is associated with a good clinical response to treatment with platinum therapies and PARP1 inhibitors [7]. A reduction of *BRCA1* expression can be a result of a deleterious germ-line variant combined with the loss of the wild type allele in the tumour [8] an idiopathic somatic mutation, that lead to mutational inactivation of both alleles [9], or by mechanisms unrelated to the occurrence of a somatic mutation, such as the inhibition of p53 protein, promoter hypermethylation or high NBR2 gene expression [10]. Recent studies demonstrated that microRNA (miRNA) can decrease BRCA1 expression by targeting the 3’UTR region of the gene [11].

MiRNAs are small non-coding RNAs, composed of 20–27 nucleotides [12], which regulate the expression of other genes and are involved in important biological processes, such as development, differentiation, apoptosis and proliferation [13]. MiRNA deregulation is strongly implicated in the pathogenesis of malignancy [14].

MiRNAs can act as an oncogene or tumour suppressor in various types of tumours. It is worth noting that miRNA regulates the response to cytostatic drugs - some of them induce resistance and others prevent it [15]. This article reviews reported relationships between various miRNAs and the response to cytostatic drugs in the treatment of breast and ovarian cancer associated with BRCA1 mutations.

## The treatment of breast and ovarian cancer in BRCA1 mutation carriers

### Therapy based on platinum drugs

Cisplatin is particularly useful in the treatment of breast and ovarian cancers associated with causative BRCA1 variants [16]. Its mechanism of action is based on the formation of cross-links between two DNA strands, and also within the same strand. This inhibits DNA replication and cell division, culminating in apoptosis [17]. Byrski et al. found that 80% of BRCA1 variant carriers, who received cisplatin as neoadjuvant treatment of breast cancer, had a good response and progression-free survival (PFS) [18].

### PARP1 inhibitor therapy

A relatively new and promising therapeutic approach for the treatment of breast cancer is the use of inhibitors of poly (ADP-ribose) polymerase (PARP), an enzyme involved in the repair of DNA breaks caused by different mechanisms, including homologous recombination. In wildtype cells, DSBs are removed by HR, but in the case of cells where BRCA1 is inactivated (by mutation or an epigenetic modification) these breaks are not repaired, resulting in DNA fragmentation and cell apoptosis [19]. The lack of properly functioning BRCA proteins can be partially compensated by PARP enzymes. If PARP inhibitors are used, the consequences of *BRCA* mutations are stronger – the possibility of repairing DBs is significantly decreased resulting in synthetic lethality [3, 4]. These data suggest the potential value of using PARP-1 inhibitors in cancer therapy. To date, Olaparib (Astra Zeneca) has been the only PARP-1 inhibitor approved for the therapeutic use. It is used in BRCA1 variant carriers – who have had a partial or complete remission (CR or PR) in the maintenance treatment of ovarian cancer sensitive to platinum drugs [11, 20]. According to the FDA recommendations, Olaparib may be used in patients who received 3 first lines of chemotherapy of cancers showing sensitivity to platins. The median progression-free survival as a result of the use of a PARP-1 inhibitor as maintenance therapy was 11.2 months compared to 4.3 months in the placebo group [21].

## MiRNA in breast and ovarian cancer associated with the BRCA1/2 mutation

MiRNA levels in various tumour types is differentially expressed (up- or down-regulated) [8]. Recent studies showed that results of the treatment with platins and PARP1 inhibitors in breast and ovarian cancers are dependant on the correlation between the increased expression levels of some miRNAs and a decrease in BRCA1 expression.

### miRNA-9

Sun et al. have searched for a group of miRNAs that target the BRCA1 3’UTR region. They used 6 algorithms to identify these miRNAs that were predicted to influence BRCA1 3’UTR. This led to the identification of 28 miRNAs that could potentially influence BRCA1 expression. Of these, miRNA-9 (miR-9) proved to be the most efficient in reducting BRCA1 activity in a luciferase reporter assay [22].

Sun et al. collected 58 patients with serous ovarian cancer (stage IIIc or IV). All of patients were treated with platinum/taxane- based chemotherapy and PFS was calculated. Samples of the tumors were used to construct the tissue microarrays. Then, they determined the levels of miR-9 expression by in-situ hybridization and the expression of BRCA1 by immuno-histochemistry.

The results of this study revealed that low BRCA1 expression and high expression of miRNA-9 was associated with platinum sensitivity and longer PFS (low vs high BRCA1 expression: median PFS = 37.3 months vs 15.2 months; high vs low miRNA-9 expression, median PFS = 26.4 months, vs 15.4 months).

These results were validated using another group of 113 ovarian tumors. Among them, 45 showed low expression of BRCA1 and 63 had elevated expression of miRNA-9. It was statistically significant that 34 tumors with low expression of BRCA1 were sensitive to platinum, as well as 43 cancers with high expression of miRNA-9.

The same group, in addition to the platinum sensitivity in patients with BRCA1 mutations, performed experiments on the response to treatment with a PARP-1 inhibitor in mice breast tumours that overexpressed miRNA-9. The results revealed that increased expression of miRNA-9 inhibits tumour growth in mice during the treatment with PARP1 AG014699 inhibitors: (PARP-1 inhibitor plus miRNA-9 agomiR: mean tumor volume = 14.3 mm3, vehicle alone: mean tumor volume = 178.8 mm3) [22].

### miRNA- 146a, miRNA-148a, miRNA-545

Gu et al. used 317 ovarian cancers to find BRCA1/2 - directed miRNAs which influences sensitivity to platinum based therapies. Therefore, they divided patients into two groups: 218 samples with wild-type BRCA1/2 and 99 samples with BRCA1/2 somatic or germline mutations. Using TCGA portal data they obtained information about miRNA expression. Then, multiple algorithms were used (f.ex. TargetScan) to identify miRNAs that potentially regulate BRCA1/2. Next, they separated the group of 218 wild-type BRCA1/2 ovarian cancers into a training and test set. Among the training set they identified 57 miRNAs targeting BRCA1/2 and between them searched for those that were associated with better overall survival (OS) in patients with wild-type BRCA1/2. Three miRNAs: miRNA-146a, miRNA-148a, miRNA-545 were identified. Next, they separated patients into miRNA related high and low- risk groups, depending on OS and PFS, using the median risk score as the cut-off. Patients from the low-risk group of miRNAs had longer median overall survival than from high-risk group (median OS = 51.9 vs 35.8 months).

Additionally, Gu et al. divided 317 ovarian cancer patients into three groups: BRCA1/2 mutation carriers, miRNA high-risk and miRNA low-risk groups. Patients with BRCA1/2 alterations had significantly longer OS than patients from the high-risk group (median OS = 49.5 vs 34.1 months). Of note, patients with BRCA1/2 mutations and those from the low-risk miRNA group had similar survival (median OS = 49.5 vs 52.2 months).

These results imply that miRNA-146a, miRNA-148a, miRNA-545 interfered with the DNA damage repair pathway by reducing BRCA1 expression [23].

In another study of miRNA-146a, Shen et al. examined 42 patients with familial breast cancer and 82 with familial ovarian cancers. Using an in vitro assay, they found that the polymorphism of G to C in miRNA-146a increased miRNA-146a expression. Furthermore, the variant allele binds more strongly to the 3’UTR BRCA1 region than allele G, which is associated with reduced BRCA1 expression [24, 25].

### miRNA – 182

Using prediction algorithms Moskwa et al. found miRNA-182 targets BRCA1 in breast cancer. In order to establish the sensitivity to IR (γ-irradiation) and PARP-1 inhibitors, they utilized up-regulated miRNA-182 in MDA-MB231 cells and downregulated miRNA-182 in 21NT cells. It was shown that MDA-MB231 cells with over-expression of miRNA-182 were significantly more sensitive to IR and PARP-1 inhibitors.

The same group also found that treatment with Olaparib (PARP-1 inhibitor) retarded growth of miR-182 expressing tumors in mice [25, 26].

### miRNA- 218

Using micro-array analysis, He et al. identified 19 deregulated miRNAs in the MCF-7/DDP cell line, including 11 down-regulated and 8 up-regulated miRNAs. One of them was miRNA-218, of which expression was most reduced in cells resistant to cisplatin in patients with BRCA1 mutations. To prove this in clinical samples, He et al. examined 85 primary breast cancer samples with different chemosensitivity to cisplatin.

No relationship was found between the level of miRNA-218 expression and the age of onset, the presence of lymph node metastases or clinical stage (He et al. 2015).

However, they found a correlation between survival rate in breast cancer and expression of BRCA1. A total of 85 patients were divided into 2 groups, depending on the levels of expression of BRCA1 and miRNA-218.

The Kaplan-Meier estimator revealed a statistically significant relationship between miRNA218, the expression of BRCA1 and survival in patients with breast cancer: [expression of miRNA-218 – survival time in months: 30.9 for low vs. 42.2 for high; expression of BRCA1- survival time in months: 41.9 for low vs. 31.5 for high expression [27].

### miRNA- 638 and 146a

Zavala et al. used 39 formalin- fixed paraffin embedded (FFPE) samples of triple negative breast cancer to find the clinical relevance of miRNA-146a and miRNA-638 in association with BRCA1 expression. The presence of BRCA1 variants was assessed. Three patients carried BRCA1 germline mutations. They divided the tumours into two groups, depending on the level of expression of miRNAs and BRCA1. It has been demonstrated that women with high expression of miRNA-638, miRNA-146a and abnormal BRCA1 had a longer overall survival in comparison to those with the low expression of these miRNAs and normally expressed BRCA1 [28].

miRNA-638 is deregulated in many different cancers [26, 29]. Therefore, Tan et al., using FFPE breast cancer samples and several human breast cancer cell lines, wanted to determine how miRNA-638 influenced sensitivity to UV-light and cisplatin in triple- negative breast cancer (TNBC). Thanks to TARGETSCAN-VERT and miRanda they revealed that miRNA-638 regulates BRCA1 expression. The FFPE samples were divided into normal tissue and breast cancer tissue and subsequently isolated RNA. Identifying that overexpression of miRNA-638 can lead to an increased susceptibility of TNBC cells to DNA damaging agents, such as UV radiation and cisplatin treatment. Although 5-fluorouracil and epirubicin also belong to the group of DNA damaging agents, no relationship was reported between an improved response to treatment with these agents and miRNA-638 levels [30].

## Conclusion

Decreased BRCA1 expression is associated with an improved response to treatment with PARP inhibitors and platinum based therapy in breast and ovarian cancer. Targeting 3’UTR region of BRCA1 specific miRNA causes down-regulation of BRCA expression.

The current literature provides data on the up-regulation of miRNAs, such as miRNA-9, miRNA-146a, miRNA-182 miRNA-218, miRNA-638 and is association with treatment response in breast and ovarian cancer when associated with the BRCA1 mutation.

The reproducibility of the effects of miRNA-146a is particularly strong because it has been shown in a several concordant studies.

In summary, a study of the levels of microRNA expression may have clinical implications for the diagnosis, prognosis and treatment of breast and ovarian cancer associated with the BRCA1 mutation.

## Abbreviations

CR: Complete remission
DSBs: Double strand breaks
FFPE: Formalin- fixed paraffin embedded
HBOC: Hereditary breast and ovarian cancer syndrome
HR: Homologous recombination
IR: γ-irradiation
miRNA: microRNA
OS: Overall survival
PARP1: Poly(ADP-ribose) polymerase
PFS: Progression-free survival
PR: Partial remission
TNBC: Triple- negative breast cancer
WHO: World Health Organization
